# The new imaging findings: “Passing spine” without kissing

**DOI:** 10.1097/MD.0000000000026191

**Published:** 2021-06-04

**Authors:** Yuichi Kasai, Tetsutaro Mizuno, Permsak Paholpak, Winai Sirichativapee, Mitsuru Fukui

**Affiliations:** a Department of Orthopaedics, Faculty of Medicine, Khon Kaen University, Khon Kaen, Thailand; b Department of Orthopaedic Surgery, Seirei Hamamatsu General Hospital, Hamamatsu; c Tetsutaro MIzunob : Seirei Hamamatsu General Hospital, Hamamatsu; d Laboratory of Statistics, Graduate School of Medicine, Osaka City University, Osaka, Japan.

**Keywords:** baastrup's disease, computed tomography, kissing spine, lumbar spinal degeneration, lumbar spine, spinous processes

## Abstract

Case-control studies by examining the lumbar spine computed tomography (CT) findings focusing on the spinous processes.

“Passing spine” was defined as a lumbar degenerative change observed on CT images. In contrast, kissing spine, which is also an image finding, has been acknowledged as an established clinical condition. Therefore, we compared the passing spine group and the kissing spine group to investigate whether the 2 groups belong to a similar disease group; this would help explain the clinical and imaging characteristics of patients with passing spine.

Previous studies have described the gradual increase in the height and thickness of the lumbar vertebral spinous processes that can occur in individuals aged >40 years, and reported that this progressive degeneration can lead to a condition termed “kissing spine.”

We examined the CT imaging of 373 patients with lumbar spinal disease and divided patients into 2 groups, the kissing spine (K) group and the passing spine (P) group, and compared the clinical (age, sex, presence/absence of lower extremity pain) and imaging data (localization of kissing or passing spine, intervertebral disc height at the level of kissing or passing spine, lumbar lordosis (LL) angle, presence/absence of vacuum phenomenon (VP) in the intervertebral discs and spondylolisthesis at the level of kissing or passing spine between the 2 groups.

Compared with patients with kissing spine, patients with passing spine had an increased incidence of lower extremity pain, lower intervertebral disc height at the level of passing spine, relatively static LL, and VP commonly observed in the intervertebral discs at the level of passing spine.

Because the clinical and imaging characteristics of patients with passing spine are different from those of patients with kissing spine, passing spine might be a pathological condition distinct from kissing spine.

## 1 Introduction

Previous studies have described the gradual increase in the height and thickness of the lumbar vertebral spinous processes that can occur in individuals aged >40 years,^[1–3]^ and reported that this progressive degeneration can lead to a condition termed “kissing spine”.^[4–5]^ We conducted a detailed inspection of computed tomography (CT) imaging of patients diagnosed with kissing spine and found that in some cases the spinous processes did not come in contact with each other; instead, the caudal portion of the upper spinous process passed beyond the cranial portion of that below it, to the caudal portion of the lower spinous process. We termed this condition “passing spine,” and the passing spine was defined as a lumbar degenerative change observed on computed tomography images.

This research study reported the clinical and imaging characteristics of passing spine for the first time. And then, kissing spine, which is also an image finding, has been acknowledged as an established clinical condition, therefore, we compared the passing spine (P) group and the kissing spine (K) group to investigate whether the 2 groups belong to a similar disease group; this would help explain the clinical and imaging characteristics of patients with passing spine. We then evaluated the clinical and imaging characteristics of patients in the P group, and discussed the pathology of lumbar spinal degeneration involving the spinous processes.

## 2 Materials and methods

The subjects of the study were 373 patients who presented with the chief complaint of lower back pain or lower back and lower extremity pain (radiating pain) and who had undergone CT examination for suspected lumbar disease at D Hospital between January 2015 and August 2017 (study duration, 2 years and 8 months). The subject population comprised 176 males and 197 females with a mean age at the time of CT of 70.1 years (range, 40–96 years) with the following suspected diagnoses: lumbar spinal canal stenosis (n = 151), spondylosis deformans (n = 73), lumbar spine fractures (n = 65), spondylolysis (n = 30), degenerative lumbar spondylolisthesis (n = 18), lumbar spine tumors (n = 16), pyogenic spondylitis (n = 6), and others (n = 14).

**Figure 1:**
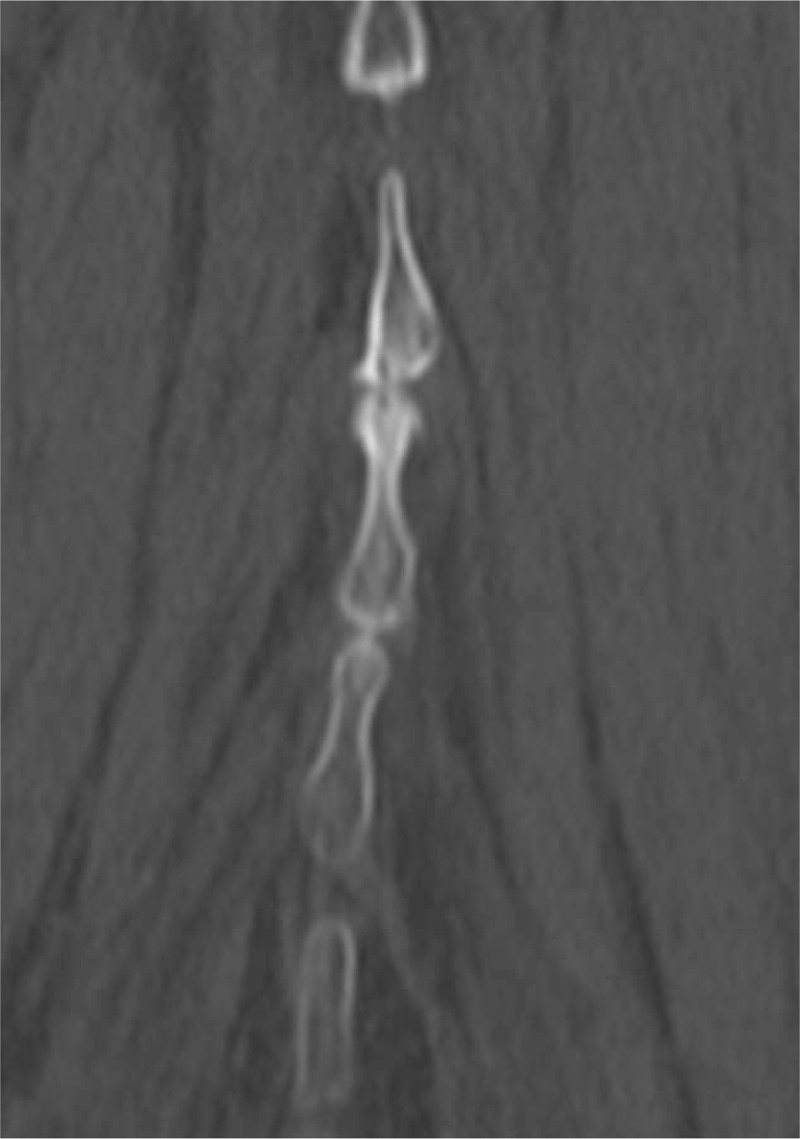
Kissing spine, spinous processes that were clearly in contact with each other.

**Figure 2:**
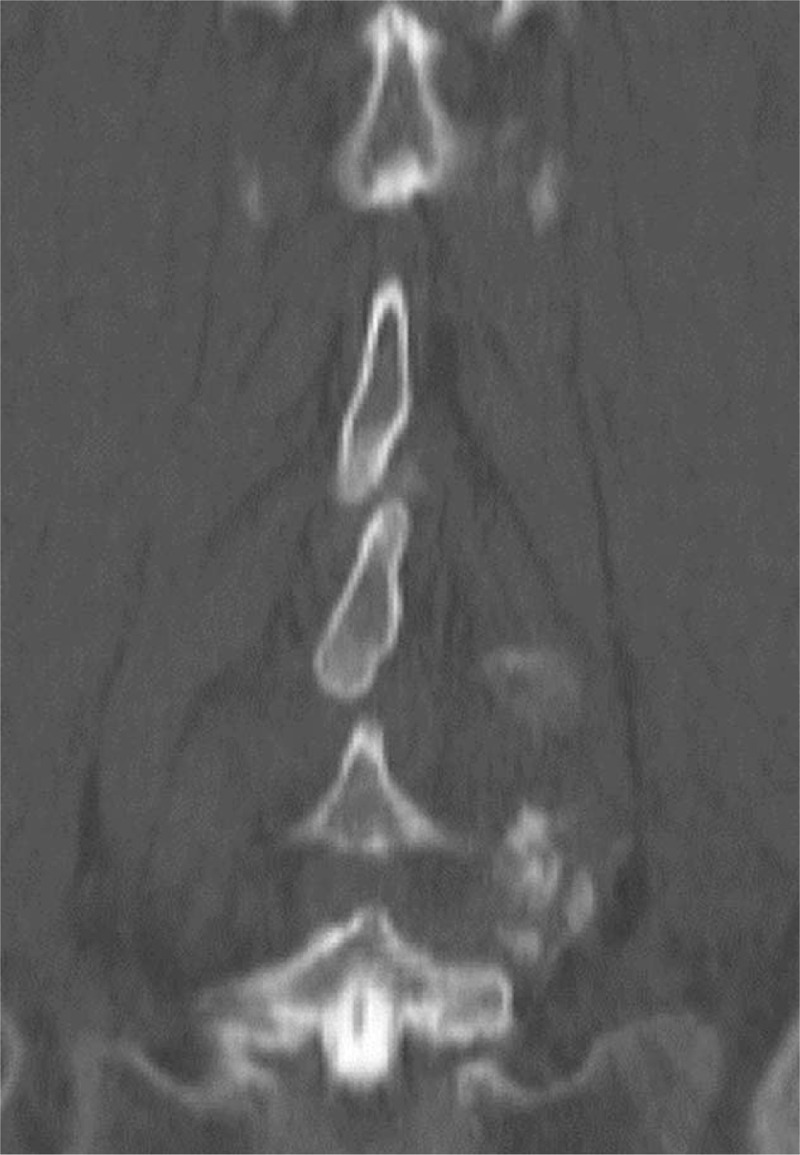
Passing spine, the caudal portion of the upper spinous process passes beyond the cranial portion of the next lower level toward the caudal portion of the lower spinous process.

In all patients, we measured the distance between the spinous processes at each intervertebral level of the lumbosacral spine (L1–2, L2–3, L3–4, L4–5, and L5–S1) on coronal CT, and identified spinous processes that were clearly in contact with each other (K group; Figure 1). Also identified were those that clearly passed beyond the cranial portion of the lower level toward the caudal portion of the lower spinous process, but did not have contact with the caudal portion of an upper spinous process (P group; Figure 2). The assessment was conducted independently by 2 spinal surgeons, YK and TM. In the event of a difference in opinion, the final decision was made by a third spinal surgeon (PP). CT was obtained using an ECLOS multislice scanner (16 slices; Hitachi Ltd., Tokyo, Japan) with multiplanar reconstruction; imaging was performed using collimation of 0.625 mm and beam pitch of 0.6. The image reconstruction interval was 0.313 mm, and patients remained in the supine position throughout scanning.

Sagittal CT images of the lumbar vertebrae were used to measure and assess the interspinous process distance and the height of the intervertebral disc (value obtained by adding the anterior and posterior vertebral disc heights and dividing by 2) at the level of kissing or passing spine, the angle between the L1 upper vertebral body and the L5 lower vertebral body (the lumbar lordosis angle [LL angle]). In the concern level of the lumbar vertebra with kissing or passing spine, the presence or absence of intervertebral vacuum phenomenon (VP) in the discs and the presence or absence of >5 mm of spondylolisthesis in the intervertebral discs were identified. These measurements and assessments were performed independently by 2 spinal surgeons, YK and TM, using measurement software supplied with the ECLOS CT system to measure the interspinous process distance, the height of the intervertebral discs and the LL angles. The mean value of the 2 measurements taken by the spinal surgeons was used as the measured value. In the event of disagreement between the 2 surgeons, a third spinal surgeon (PP) made the final decision.

The K and P groups were compared in terms of sex, age, presence/absence of lower extremity pain, localization of kissing or passing spine and the intervertebral disc height at the level of the kissing or passing spine, LL angle, presence/absence of VP in the intervertebral disc in the involved level of the lumbar vertebra at the involved level, and spondylolisthesis at the involved level.

The Mann–Whitney *U* test was used to compare age, the intervertebral disc height, and LL angle between the groups; and Fisher exact test was used to compare sex, presence/absence of lower extremity pain, localization of kissing or passing spine, presence/absence of VP in the intervertebral disc and spondylolisthesis in the concern level of the lumbar vertebra with kissing or passing spine. The level of statistical significance was established as *P* < .05.

This study was approved by the Ethic committee for human research, Khon Kaen University (Approval no. HE631013).

## 3 Results

Of a total of 373 patients, 171 intervertebral levels in 73 patients (19.6%) were assessed as having kissing spine and 19 intervertebral levels in 19 patients (5.1%) were assessed as having passing spine; no levels presented as both. The K group (n = 73) comprised 41 males and 32 females with a mean age of 73.8 years (range, 49–96 years), and the P group (n = 19) comprised 7 males and 12 females with a mean age of 69.7 years (range, 40–90 years). All interspinous process distances including 171 intervertebral levels in the K group and 19 intervertebral levels in the P group were zero or less than 1 mm on sagittal CT. Inter-rater agreement of the assessment of kissing spine and passing spine was consistent (in 167 of 171 intervertebral spaces in the K group and in all 19 intervertebral spaces in the P group), resulting in an overall agreement rate of 97.9%.

In the K group, the level showing kissing or passing spine was localized as follows: L1–2, n = 7; L2–3, n = 19; L3–4, n = 28; L4–5, n = 56; and L5–S1, n = 61. In the P group, the levels were as follows: L1–2, n = 0; L2–3, n = 1; L3–4, n = 6; L4–5, n = 7; and L5–S1, n = 5. Lower extremity pain was present in 46 of 73 patients in the K group and in 17 of 19 patients in the P group. Mean intervertebral disc height at the level of kissing or passing spine was 5.7 mm (range, 1–12 mm) in the K group and 3.4 mm (range, 1–7 mm) in the P group. The mean LL angle was 16.6° (range, 2–58°) in the K group and 27.1° (range, 15–69°) in the P group. VP was identified in the intervertebral disc at the involved level in 56 levels (32.7%) discs in the K group and in 13 levels (68.4%) discs in the P group. And, the spondylolisthesis at the involved level was identified in 14 levels (8.2%) in in the K group and in 3 levels (15.8%) in the P group. Regarding inter-rater agreement, assessment of VP in the intervertebral discs and spondylolisthesis was consistent in 65 of 69 intervertebral discs identified as having VP, and in 15 of 17 involved levels identified as having spondylolisthesis. The rate of agreement was 96.8%.

The results of comparison of the clinical and imaging characteristics between the 2 groups (Table 1) were as follows: age, *P* = .1179; sex, *P* = .1971; presence/absence of lower extremity pain, *P* = .0289; localization of kissing or passing spine, *P* = .27810; intervertebral disc height at the level of kissing or passing spine, *P* = .0313; LL angle, *P* = .0468; presence/absence of VP in the intervertebral discs at the involved level, *P* = .0044; and presence/absence of spondylolisthesis at the involved level, *P* = .2860. Thus, compared with the K group, lower extremity pain occurred significantly more frequently (*P* < .05), the LL angle was significantly greater (*P* < .05), intervertebral height at the level of kissing or passing spine was significantly smaller (*P* < .05), and VP was seen more frequently in the intervertebral discs at the involved level (*P* < .01) in the P group. No significant difference was found between the 2 groups in terms of age, sex, localization of kissing or passing spine, or the presence/absence of spondylolisthesis at the involved level.

**Table 1:** Results of comparison of the clinical and imaging characteristics between Kissing spine group and Passing spine group.

	Kissing spine group(73 cases, 171 levels)	Passing spine group(19 cases, 19 levels)	Significant differences
mean age	73.8 yr(49–96)	69.7 yr(40–90)	n.s.(*P* = .1179)
sex	41 males, 32 females	7 males, 12 females	n.s.(*P* = .1971)
presence of lower extremity pain	46 cases	17 cases	*P* < .05(*P* = .0289)
localization of kissing or passing spine	L1–2; 7 levelsL2–3; 19 levelsL3–4; 28 levelsL4–5; 56 levels L5–S1; 61 levels	L2–3; 1 level L3–4; 6 levelsL4–5; 7 levels L5–S1; 5 levels	n.s. (*P* = .2781)
intervertebral disc height at the level of kissing or passing spine	mean 5.7 mm(1–12 mm)	mean 3.4 mm(1–7 mm)	*P* < .05(*P* = .0313)
mean LL	16.6°(2–58°)	27.1°(15–69°)	*P* < .05(*P* = .0468)
presence of VP at the level of kissing or passing spine	56 levels(32.7%)	13 levels(68.4%)	*P* < .01(*P* = .0044)
presence of spondylolisthesis at the level of kissing or passing spine	14 levels(8.2%)	3 levels(15.8%)	n.s.(*P* = .2860)

## 4 Discussion

Kissing spine syndrome, or contact between adjacent spinous processes, is also called Baastrup's disease,^[5]^ and often develops in the lower lumbar spine in the elderly and may result in lower back pain.^[6–8]^ Regarding the frequency of kissing spine, Kwong et al^[9]^ examined the abdominal CT images of 1008 patients and identified kissing spine in 413 (41.0%). Maes et al^[10]^ identified the presence of a bursa between the lumbar spinous processes on MRI as a characteristic of kissing spine syndrome in 44 of 539 cases (8.2%). Because we did not administer local anesthesia injections for kissing spinal lesions or perform MRI imaging in all of the present patients, we could not make a final definitive diagnosis of kissing spine syndrome;^[11,12]^ however, our survey of CT lumbar spine images revealed that kissing spine was identifiable on CT in 73/373 patients (19.6%), which confirmed that kissing spine is a relatively common lumbar image finding in the elderly.

In this study, we propose the term “passing spine” for the image finding of contact between adjacent spinous processes in which the caudal portion of the upper spinous process clearly passes beyond the cranial portion of the lower level toward the caudal portion of the lower spinous process, but without contact. Passing spine was observed in 19/373 patients (5.1%) and showed characteristics such as a tendency of lower extremity pain, lower intervertebral height at the level of passing spine, relatively static LL, and VP in the intervertebral discs at the involved level. These characteristic findings are different to those in kissing spine, and indicate that patients in the P group are more likely to develop degenerative intervertebral discs and to be susceptible to neurological symptoms.

Degenerative changes in the lumbar spine due to aging cause the gradual loss of physiological LL due to decreasing height of the intervertebral discs, vertebral bodies, and anterior spine elements; and hypertrophy of the spinous processes and posterior spine elements.^[13–15]^ Furthermore, hyper-extension of the spine in patients with lumbar spinal disease causes bulging of the anterior intervertebral discs and buckling of the ligamenta flava, compressing the dura mater tube and nerve roots, which can result in a neurologic manifestation.^[16–18]^ Of the present patients, lower extremity pain was reported less frequently by those in the K group, for the possible reason that the kissing spinous processes may have physically prevented hyper-extension of the lumbar spine and led to a “blocking” effect, preventing compression of the dura mater tube and nerve roots.^[19–23]^ In contrast, in the patients in the P group, a severely degenerated intervertebral disc may have caused the vertebral body to rotate either right or left, resulting in displacement of the original alignment of the spinous processes. As displacement progressed, the adjacent spinous processes would not directly contact each other; instead, the caudal portion of the upper spinous process was seen clearly to pass beyond the cranial portion of the lower level and towards the caudal portion of the lower spinous process. Accordingly, in patients with passing spine, there is no blocking effect on the lower intervertebral disc height, and hyper-extension of the lumbar spine does not function to provide this protective function; thus, lower extremity pain developed more frequently in the P group. However, this is still an assumption requiring further inspection of dynamic images and biomechanical examination for verification.

There are some limitations of the present study:

1. a small number of cases were included, especially in the P group;
2. the presence/absence of kissing spine was determined from the CT findings and static images alone, and dynamic elements were not considered;
3. we did not conduct a detailed investigation of degeneration of the intervertebral discs and joints;
4. we did not investigate patients who did not fit the criteria for admission to the K or P groups;
5. we did not investigate progression of degeneration on the images over time; and
6. we did not evaluate the clinical data using such as the Back Pain Evaluation Questionnaire of the Japanese Orthopaedic Association.

Therefore, we plan to conduct further research to clarify the causes and pathology of passing spine that will include detailed investigation of the clinical and imaging characteristics of this condition in a larger number of cases, evaluation of the findings of functional imaging with plain X-rays and MRI imaging, and examination of change in the imaging findings over time. In addition, we would like to investigate the clinical and imaging characteristics of cases of progressive degeneration that do not fall the category of kissing spine or passing spine.

In conclusion, this study is the first to report the imaging findings of “passing spine,” in which the caudal portion of the upper spinous process passes beyond the cranial portion of the next lower level toward the caudal portion of the lower spinous process, by examining the lumbar spine CT findings focusing on the spinous processes. Compared with patients with kissing spine, patients with passing spine had an increased incidence of lower extremity pain, lower intervertebral disc height at the level of passing spine, relatively static LL, and VP commonly observed in the intervertebral discs at the involved level. Because the clinical and imaging characteristics of patients with passing spine is different from those of patients with kissing spine, passing spine might be a pathological condition distinct from kissing spine.

## Author contributions

**Conceptualization:** Yuichi Kasai.

**Data curation:** Yuichi Kasai, Tetsutaro Mizuno.

**Methodology:** Yuichi Kasai, Tetsutaro Mizuno, Permsak Paholpak, Mitsuru Fukui.

**Project administration:** Permsak Paholpak.

**Software:** Mitsuru Fukui.

**Supervision:** Winai Sirichativapee.

**Validation:** Mitsuru Fukui.

**Writing – original draft:** Yuichi Kasai, Tetsutaro Mizuno.

**Writing – review & editing:** Permsak Paholpak, Winai Sirichativapee.

## References

[1] Scapinelli R. Morphological and functional changes of the lumbar spinous processes in the elderly. Surg Radiol Anat 1989;11:129–33. 2763004 10.1007/BF02096469

[2] Paholpak P Wang Z Sakakibara T. An increase in height of spinous process is associated with decreased heights of intervertebral disc and vertebral body in the degenerative process of lumbar spine. Eur Spine J 2013;22:2030–4. 23546689 10.1007/s00586-013-2764-y PMC3777063

[3] Aylott CE Puna R Robertson PA. Spinous process morphology: the effect of ageing through adulthood on spinous process size and relationship to sagittal alignment. Eur Spine J 2012;21:1007–12. https://doi.org/10.1007%2Fs00586-011-2029-6 21959943 10.1007/s00586-011-2029-6 PMC3337914

[4] Brailsford JF. Deformities of the lumbosacral region of the spine. Br J Surg 1929;16:562–627.

[5] Baastrup C. On the spinous processes of the lumbar vertebrae and the soft tissues between them, and on pathological changes in that region. Acta Radiol 1933;14:52–4.

[6] Filippiadis DK Mazioti A Argentos S. Baastrup's disease (kissing spines syndrome): a pictorial review. Insights Imaging 2015;6:123–8. 25582088 10.1007/s13244-014-0376-7 PMC4330238

[7] Alonso F Bryant E Iwanaga J Chapman JR Oskouian RJ Tubbs RS. Baastrup's disease: a comprehensive review of the extant literature. World Neurosurg 2017;101:331–4. 28192272 10.1016/j.wneu.2017.02.004

[8] Schwartz RH Urits I Viswanath O. Extensive degeneration of vertebral body leading to baastrup's disease: a radiographic review of an image. Pain Ther 2019;8:285–7. 31686398 10.1007/s40122-019-00138-3 PMC6857106

[9] Kwong Y Rao N Latief K. MDCT findings in Baastrup disease: disease of normal feature of the aging spine. Am J Roentgenol 2011;196:1156–9. 21512085 10.2214/AJR.10.5719

[10] Maes R Morrison WB Parker L. Lumbar interspinous bursitis (Baastrup disease) in a symptomatic population: prevalence on magnetic resonance imaging. Spine (Phila Pa 1976) 2008;33:E211–5. 18379391 10.1097/BRS.0b013e318169614a

[11] Koda M Mannoji C Murakami M. Baastrup's disease is associated with recurrent of sciatica after posterior lumbar spinal decompressions utilizing floating spinous process procedures. Asian Spine J 2016;10:1085–90. 27994785 10.4184/asj.2016.10.6.1085 PMC5164999

[12] Kerroum A Laudato PA Suter MR. The steps until surgery in the management of Baastrup's Disease (kissing spine syndrome). J Surg Case Rep 2019;2019:rjz194 10.1093/jscr/rjz194 31275550 PMC6598627

[13] Niosi CA Oxland TR. Degenerative mechanics of the lumbar spine. Spine J 2004;4:202S–S208. 15541668 10.1016/j.spinee.2004.07.013

[14] Sparrey CJ Bailey JF Safaee M. Etiology of lumbar lordosis and its pathophysiology: a review of the evolution of lumbar lordosis, and the mechanics and biology of lumbar degeneration. Neurosurg Focus 2014;36:E1 10.3171/2014.1.FOCUS13551 24785474

[15] Chun SW Lim CY Kim K Hwang J Chung SG. The relationships between low back pain and lumbar lordosis: a systematic review and meta-analysis. Spine J 2017;17:1180–91. 28476690 10.1016/j.spinee.2017.04.034

[16] Postacchini F Gumina S Cinotti G. Ligamenta flava in lumbar disc herniation and spinal stenosis. Light and electron microscopic morphology. Spine (Phila Pa 1976) 1994;19:917–22. 8009349 10.1097/00007632-199404150-00009

[17] Chung SS Lee CS Kim SH Chung MW Ahn JM. Effect of low back posture on the morphology of the spinal canal. Skeletal Radiol 2000;29:217–23. 10855470 10.1007/s002560050596

[18] Hansson T Suzuki N Hebelka H Gaulitz A. The narrowing of the lumbar spinal canal during loaded MRI: the effects of the disc and ligamentum flavum. Eur Spine J 2009;18:679–86. 19277726 10.1007/s00586-009-0919-7 PMC3234003

[19] Inufusa A An HS Lim TH. Anatomic changes of the spinal canal and intervertebral foramen associated with flexion-extension movement. Spine 1996;21:2412–20. 10.1097/00007632-199611010-00002 8923625

[20] Willen J Danielson B Gaulitz A Niklason T Schönström N Hansson T. Dynamic effects on the lumbar spinal canal: axially loaded CT-myelography and MRI in patients with sciatica and/or neurogenic claudication. Spine 1997;22:2968–76. 9431634 10.1097/00007632-199712150-00021

[21] Schmid MR Stucki G Duewell S Wildermuth S Romanowski B Hodler J. Changes in cross-sectional measurements of the spinal canal and intervertebral foramina as a function of body position: in vivo studies on an open-configuration MR system. Am J Roentgenol 1999;172:1095–102. 10587155 10.2214/ajr.172.4.10587155

[22] Fujiwara A An HS Lim TH Haughton VM. Morphologic changes in the lumbar intervertebral foramen due to flexion-extension, lateral bending, and axial rotation: an in vitro anatomic and biomechanical study. Spine 2001;26:876–82. 11317109 10.1097/00007632-200104150-00010

[23] Wiseman CM Lindsey DP Fredrick AD Yerby SA. The effect of an interspinous process implant on facet loading during extension. Spine 2005;30:903–7. 15834334 10.1097/01.brs.0000158876.51771.f8

